# *dLp/HDL-BGBP* and *MTP* Cloning and Expression Profiles During Embryonic Development in the Mud Crab *Scylla paramamosain*

**DOI:** 10.3389/fphys.2021.717751

**Published:** 2021-08-19

**Authors:** Xianyuan Zeng, Liang Lyu, Dousha Zhao, Jinying Zhong, Yan Feng, Haifu Wan, Chunyang Li, Ziping Zhang, Yilei Wang

**Affiliations:** ^1^Key Laboratory of Healthy Mariculture for the East China Sea, Ministry of Agriculture and Rural Affairs, Fisheries College, Jimei University, Xiamen, China; ^2^School of Life Sciences, Ningde Normal University, Ningde, China; ^3^Department of Student Affairs, Ningde Normal University, Ningde, China; ^4^Key Laboratory of Marine Biotechnology of Fujian Province, Institute of Oceanology, College of Animal Science, Fujian Agriculture and Forestry University, Fuzhou, China

**Keywords:** *dLp/HDL-BGBP*, *MTP*, lipid transfer, embryo, mud crab, decapod

## Abstract

Lipids are the main energy source for embryonic development in oviparous animals. Prior to the utilization and catabolism, lipids are primarily transported from the yolk sac to embryonic tissues. In the present study, cDNA encoding a circulatory large lipid transfer protein (LLTP) superfamily member, the precursor of large discoidal lipoprotein (dLp) and high-density lipoprotein/β-1,3-glucan-binding protein (HDL-BGBP), named *dLp/HDL-BGBP* of 14,787 bp in length, was cloned from the mud crab *Scylla paramamosain*. *dLp/HDL-BGBP* was predicted to encode a 4,831 amino acids (aa) protein that was the precursor of dLp and HDL-BGBP, which were both detected in hemolymph by liquid chromatography–mass spectrometry (LC-MS/MS) analysis. For the intracellular LLTP, three *microsomal triglyceride transfer protein* (*MTP*) cDNAs of 2,905, 2,897, and 3,088 bp in length were cloned from the mud crab and were predicted to encode MTP-A of 881 aa, MTP-B of 889 aa, and MTP-C of 919 aa, respectively, which were different merely in the N-terminal region and shared an identical sequence of 866 aa. During embryonic development, the expression level of *dLp/HDL-BGBP* consecutively increased from the early appendage formation stage to the eye pigment-formation stage, which indicated that HDL-BGBP is probably the scaffolding protein for yolk lipid. For the *MTP* gene, *MTP-C* accounted for ~70% of *MTP* mRNA from the blastocyst stage to the nauplius stage, as well as the pre-hatching stage; *MTP-C* and *MTP-A* expression levels were comparable from the early appendage formation stage to the late eye pigment-formation stage; *MTP-A* was extremely low in blastocyst and gastrula stages; *MTP-B* was expressed at a relatively low-level throughout embryo development. The variations in the expression profiles among *MTP* transcripts suggested that *MTP* might play roles in the lipid droplet maturation and lipoprotein assembly during embryonic development.

## Introduction

Lipids are the main energy source for embryonic development and are stored in yolk sac-forming droplets in oviparous animals. The yolk lipids that accumulate originally in oocytes are derived from tissues of fat absorption and/or storage during ovarian maturation (Schneider, 2016; Fruttero et al., 2017; Price, 2017; Quinlivan and Farber, 2017). The yolk lipid content and composition, to a large extent, determine egg quality, offspring survival, and reproduction performance, of which the root cause is the nutrient intake and maternal investment (Wouters et al., 2001; Harlioglu and Farhadi, 2017; Lubzens et al., 2017). Beyond nutrient supply, increasing evidence reveal that lipids play critical roles in many biological processes (Welte, 2015a,b; Welte and Gould, 2017). For example, excess histones are essential for chromatin assembly in early *Drosophila* embryos, whereas the free histones are toxic and the massive histones are anchored to lipid droplets (Li et al., 2012). The histones in lipid droplets are also released and dedicated to antibacterial defense when bacteria invade (Anand et al., 2012). Although specific roles of lipid droplets and associated mechanisms in the embryo remain to be determined, lipid transfer from the yolk sac to embryonic tissues is undoubtedly the prerequisite of supply and function (Heras et al., 2000; Speake and Thompson, 2000; Yamahama et al., 2008; Miyares et al., 2014; Schneider, 2016). Therefore, yolk lipid transfer is extremely vital for embryonic development in oviparous animals.

The large lipid transfer proteins (LLTPs) are the most well-known scaffolding proteins for lipid transfer in animals. The LLTPs have conserved lipoprotein N-terminal domain (LPD_N) architecture. In phylogenetic trees, the superfamily members have been divided into three groups, apolipoprotein B-like proteins (APOs), vitellogenin-like proteins (VTGs), and microsomal triglyceride transfer protein-like proteins (MTPs) (Smolenaars et al., 2007; Hayward et al., 2010; Wu et al., 2013). The APO family members are generally endued with an outstanding lipid carrying capacity, such as apolipoprotein B (apoB) in vertebrates and apolipophorin-II/I (apoLp-II/I) in insects, as compared to that in the other two family members (Smolenaars et al., 2007). The VTG family members mainly function as the amino acid source for embryonic development (Li and Zhang, 2017). The MTP family members are intracellularly dedicated to lipoprotein assembly of APO and VTG members with lipid molecules (Hussain et al., 2003; Sellers et al., 2005). During embryonic development, vertebrates, and even mammalian embryos in the earliest stage, share a similar process that lipids are internalized by the endodermal epithelial cells of the yolk sac. Here, apoB is lipidated forming very low-density lipoproteins, which are mediated by MTP (Madsen et al., 2004; Bauer et al., 2013; Schneider, 2016; Quinlivan and Farber, 2017). The mechanism of lipid transfer in the yolk sac appears to be consistent with that in the liver and intestinal tract in vertebrates. In the contrast, information on lipid transfer in invertebrates during embryonic development is still scarce.

In decapod species, distinctive characteristics have been described in the structure and function of LLTPs (Avarre et al., 2007; Wu et al., 2013; Hoeger and Schenk, 2020). Specifically, decapodan “vitellogenin” is not a member of the VTG family but *de facto* belongs to the APO family, for which the name was consequently suggested to be apolipocrustacein (apoCr) (Avarre et al., 2007). In addition to being the major egg yolk protein, apoCr is also the ovary-specific lipid carrier during the vitellogenesis process (Subramoniam, 2011; Zeng et al., 2021). Another APO family member in decapodan species is the common precursor of large discoidal lipoprotein (dLp) and high-density lipoprotein/β-1,3-glucan-binding protein (HDL-BGBP), named dLp/HDL-BGBP (Stieb et al., 2014). The latter is regarded as the major circulatory lipid carrier, which is still not understood given the absence of the LPD_N domain that is believed to be the main lipidation site, whereas the former is still unknown in function and has only been detected in hemolymph of *Astacus leptodactylus* (Stieb et al., 2008, 2014; Hoeger and Schenk, 2020). Recently, a decapodan *MTP* cDNA was identified from mud crabs, *Scylla paramamosain*, and it appears to be conserved in the structure and function compared to that in other animals (Zeng et al., 2021). During embryonic development, apoCr and dLp/HDL-BGBP are both detected in embryos with yolk sacs of *Macrobrachium borellii* and *Callinectes sapidus*, and the protein level of the former decreases consecutively, whereas the concentration of the latter is consecutively increased (Walker et al., 2003, 2006; García et al., 2008), which imply that the scaffolding protein of yolk lipids is dLp/HDL-BGBP in the embryo. However, information on dLp/HDL-BGBP-containing lipoprotein assembly is still unknown during embryonic development.

*Scylla paramamosain* is a commercially important species in South China, and crab aquaculture has continued increasing during the last few decades. Nonetheless, seeds for crab culture are mainly supplied by a wild-catch method because of limited seed production in which hatchery technology is still in its infancy. To develop reliable hatchery technology, a comprehensive understanding of embryo development is required for the mud crab. Transcriptome data of *S. paramamosain* embryos in our lab implied that dLp/HDL-BGBP and MTP might be involved in lipid transfer during embryonic development. To understand their roles in yolk lipid transfer, the present study aimed to (1) clone *dLp/HDL-BGBP* and *MTP* cDNA in *S. paramamosain*, (2) detect the occurrence of dLp and HDL-BGBP in hemolymph, and (3) characterize the expression of *dLp/HDL-BGBP* and *MTP* during embryonic development.

## Methods and Materials

### Animals and Sampling

The female mud crabs in the present study were purchased from a seafood retailer in Zhaoan, Zhangzhou, Fujian, China. Three Crabs in the late vitellogenic stage were anesthetized in ice for ~10–20 min prior to sampling (Bao et al., 2015). Hemolymph drops were collected into 15 ml Falcon-type tubes containing 0.1 volumes of EDTA anticoagulant (50 mM EDTA, 225 mM NaCl, and pH 7.4). Hemolymph samples were centrifuged for 10 min at 5,000 × *g* at 4°C to obtain plasma. Subsequently, crabs were dissected and the hepatopancreas was sampled immediately.

In order to collect embryo samples, fertilized crabs were housed in a marine aquaculture facility of Jimei University, in which the water temperature was 25–28°C and water salinity was ~25 ppt (Liao et al., 2020). The crabs were fed daily with live shellfish at *ca*. 3% of their body weight. After 5 days of acclimation, unilateral eyestalk ablation was performed with sterilized tweezers to induce spawning. The embryos consecutively collected from four berried crabs in the present study were divided into nine developmental stages (Chen, 2005), such as blastocyst (I), gastrula (II), nauplius (III), early and late appendage formation (IV–V), early, middle, and late eye pigment formation (VI–VIII), and prehatching (IX). All samples were frozen in liquid nitrogen and stored at −80°C until the following analyses.

### RNA Isolation, cDNA Synthesis, and cDNA Cloning

To clone *dLp/HDL-BGBP* and *MTP* cDNAs, total RNA was isolated from hepatopancreas samples using an RNA extraction kit (Promega, Shanghai, China) following the instructions of the manufacturer. The quality and quantity of total RNA were determined by 1% agarose gel electrophoresis and with a Nanodrop 2000 spectrometer (Thermo Scientific, Wilminton, Delaware, USA), respectively. The total RNA was treated with DNase I to remove contaminating genomic DNA and subjected to reverse transcription using random primers, dNTP mix, and M-MLV (Promega, Shanghai, China) for the first-strand cDNA. The cDNA fragments of *dLp/HDL-BGBP* and *MTP* were amplified using a set of primer pairs (Tables 1, 2), which were designed based on our transcriptome data (PRJNA694319).

**Table 1:** Primers used for *dLp/HDL-BGBP* cloning in the present study.

**Name**	**Forward primer (5'-3')**	**Reverse primer (5'-3')**
*dLp/HDL-BGBP-1*	CTGTCACGACCAACACTGCC	CATGCCCATTATGTCCAAGC
*dLp/HDL-BGBP-2*	CTGCTGGACACCCACTACTC	TTTCACCCACTTGGACCTCT
*dLp/HDL-BGBP-3*	ATTCCCTCCTCGTCTTGCTT	ATTGTGCCCTTGATGTTCCT
*dLp/HDL-BGBP-4*	ACAGAGGTCCAAGTGGGTGA	CGTGTAGTGTTCGTTGAGGG
*dLp/HDL-BGBP-5*	TCACGCACAAGATTGAGAAC	AGGCGAGGCTGAGATTA
*dLp/HDL-BGBP-6*	CAAGTACGAGGCAAAGGTGA	GCCGAGGAAGTTGTAGGAAG
*dLp/HDL-BGBP-7*	ACGCAAGATTGAGGTGACGG	ATGGTGGCATCCAGGACAGA
*dLp/HDL-BGBP-8*	GTGCGGTGCGACGACTTCCA	GAGGCGGTTGAGCCCTGTGA
*dLp/HDL-BGBP-9*	GAGTCTGTCCTGGATGCC	GTTCGGTGTTCTGGGTC
*dLp/HDL-BGBP-10*	ACGAGGGTATGTGGAAGAGG	ACGGTGGATGTGGAAGTTGG
*dLp/HDL-BGBP-11*	GAGGGTATGTGGAAGAGGAA	GTTGTTGTTGTTGCGGAAGT
*dLp/HDL-BGBP-12*	ACCTTGGCAATGATGGAA	GAAGTCTGCTGTGGCGTA
*dLp/HDL-BGBP-13*	ACCACGACTTCCGCAACAAC	ACCTCAGCACGCATCACCAC
*dLp/HDL-BGBP-14*	GCCGACAAGTCTCAGTTC	CCAGTAGGGATGTAGGGA
*dLp/HDL-BGBP-15*	GAGGTCCGAGTTGATGAG	GACAGTGAGGTTGCCAGT
*dLp/HDL-BGBP5'-gsp*		GGCGAGTCTGTGTTGTAGGAG
*dLp/HDL-BGBP5'-outer*		GAGAGGTCCTTCTGCTTGGTGACGGTGA
*dLp/HDL-BGBP5'-inner*		TCGCTTCAGGTTGAGCACCCAAATGTCC
*dLp/HDL-BGBP3'-outer*	TCCCTACATCCCTACTGGGCTGGTCAA	
*dLp/HDL-BGBP3'-inner*	ACTATCCGTGCCACAGTCACCACCCCT	

**Table 2:** Primers used for *MTP* cloning in the present study.

**Objective**	**Name**	**Forward primer (5'-3')**	**Reverse primer (5'-3')**
Identical region	*MTP-1*	GCAGCCTTGGACCACGAT	TGCTTCCTTGCCTTCACCCT
	*MTP-2*	CACAAGTCATCAAGGGTAGA	TATCCAGTAGTTGAGGCAGA
	*MTP-3*	GAAGGCAAGGAAGCAAAC	AGTGAAAGGCTGATGAGGTA
	*MTP-4*	GCAGCCATAGCCTTTACTCG	ATCATCCACAGCATCCTCCC
	*MTP-5*	GCTGGGAAGATTATGGGATT	GCTGTCAACACGAGCCACT
	*MTP-6*	GTTTGCGGGTGGGCTTAGTG	GTGTTTGGCTGGACCATTTT
	*MTP-7*	AGCCAACGCAGGGATAG	GGGGACAGCCTCTTCTTA
	*MTP-3'-outer*	TTGGCAGGACAGATTCAGGT	
	*MTP-3'-inner*	CATTCCTGGCACCAACTACA	
*MTP-A* specific	*MTP-A-5'-gsp*		GGCACTGACACTACATTCTGGC
	*MTP-A-5'-outer*		ACCGACAACTTGGGGGTCACCACCGTAA
	*MTP-A-5'-inner*		GTAGAAACTGGTAGGGGCTGTGGCTCAT
*MTP-B* specific	*MTP-B*	GCAGCGTAAACCTTTGCCAT	ACACATATAGCGTCCCCACC
*MTP-C* specific	*MTP-C-5'-gsp*		GGGGGTCACCACCGTAAGC
	*MTP-C-5'-outer*		GGTAGGAGGAGAGCAGCACAAAGGCAT
	*MTP-C-5'-inner*		CAGCACGGGGGTCGTCACAGCACTCAC

To obtain cDNA untranslated regions (UTRs), 5′ and 3′ rapid amplification of cDNA end (RACE) reactions were conducted using 5′ (Sangon Biotech, China) and 3′ RACE kits (Clontech, USA), according to the protocols of the manufacturer. Briefly, the first-strand cDNA was derived from total RNA using a gene-specific primer (GSP) for 5′ RACE PCR and a 3′ oligo(dT) primer in the kit for 3′ RACE PCR. Two-round touchdown PCR reactions were run as five cycles at 94°C for 30 s and 72°C for 3 min; 5 cycles at 94°C for 30 s, 70°C for 30 s, and 72°C for 3 min; 25 cycles at 94°C for 30 s, 68°C for 30 s, and 72°C for 3 min; and a final extension at 72°C for 10 min. Two gene-specific primers (outer primer for first round PCR, inner primer for second round PCR) were paired with round-specific adapter primers in the RACE kits. All PCR primers for gene cloning are listed in Tables 1, 2.

### Analysis of Liquid Chromatography–Mass Spectrometry (LC-MS/MS)

The total protein concentration of plasma samples was determined with a BCA protein assay kit P0012 (Beyotime Institute of Biotechnology, Shanghai, China). Subsequently, plasma samples (*ca*. 20 μL) were reduced, alkylated, and digested to tryptic peptides (Yang et al., 2015). The tryptic peptides were desalted (C18 Cartridges, bed I.D. 7 mm, 3 ml volume), concentrated (Vacufuge Concentrator, Eppendorf), and diluted into 40 μl of formic acid (0.1% v/v).

The tryptic peptides (*ca*. 4 μL) were separated on an EASY-nLC 1200 liquid chromatography system (Thermo Fisher Scientific). Briefly, the peptides were eluted in an Easy C18 analytical column (150 mm × 75 μm, 3 μm particle size), using solvent A (0.1% formic acid) and B (80% acetonitrile containing 0.1% formic acid). The gradient elution ran at 300 nl/min flow rate, during which solvent B was increased from 5 to 8% over 2 min, 8 to 23% over 88 min, 23 to 40% over 10 min, and 40 to 100% over 8 min and maintained at 100% for 12 min. The separated peptides were injected into a Q Exactive mass spectrometer (MS; Thermo Fisher Scientific) operating in positive ion mode. For the full MS scan (m/z 300–1,800), precursor ions were isolated using the data-dependent acquisition mode with a resolution of 60,000 at m/z 200, automatic gain control target at 3e6, and maximum injection time at 50 ms. The 20 ions with the highest intensity were selected for the MS/MS scan, with a resolution of 15,000 at m/z 200 and a maximum injection time at 50 ms, and fragmented in the instrument ion trap at normalized collision energy (28 eV).

The MS data were processed with MaxQuant (version 1.6.0.16, Max Plank Institute for Biochemistry, Germany) and searched against the UniProt database of Portunidae protein sequences downloaded on January 21, 2021. The search results were filtered using the parameters such as main search tolerance at 4.5 ppm, first search peptide tolerance at 20 ppm, MS/MS tolerance at 20 ppm, carbamidomethylation of cysteine as a fixed modification, oxidation of methionine and acetylation of the protein N-term as variable modifications, enzyme trypsin, and maximum missed cleavages of 2. The false discovery rate against a reversed decoy database was <0.1% for proteins.

### Bioinformatics Analysis

To characterize protein structure, SignalP 5.0, ProP 1.0, SMART, and Compute pI/Mw programs were used to predict signal sequence, furin cleavage site, conserved domain architecture, and molecular mass, respectively (Duckert et al., 2004; Petersen et al., 2011; Letunic and Bork, 2018). To construct the phylogenetic tree, maximum likelihood analysis was performed using PhyML 3.0 with the LG substitution model selected by MEGA X based on the Akaike information criterion (Le and Gascuel, 2008), using aligned LPD_N domain sequences of decapodan LLTPs. These LLTPs were deduced from genes with cDNA sequence support published in the NCBI GenBank. In addition, *dLp/HDL-BGBP* and *MTP* retrieved from transcriptome data of decapodan species were also included. The detailed information of decapodan LLTPs is provided in the Supplementary Table.

### Real-Time Quantitative PCR

To characterize *dLp/HDL-BGBP* and *MTP* expression during embryonic development, the expression level in different development stages was determined by real-time quantitative PCR (qPCR). The qPCR was performed on a LightCycler 480 system (Roche, Switzerland) using SYBR Green PCR MasterMix. The qPCR reaction program comprised an initial step at 95°C for 30 s, followed by 40 repeated cycles of heating at 95°C for 30 s, annealing at 60°C for 30 s, and a final extension at 72°C for 30 s. Each group included four biological replicates and each run was repeated three times. The relative expression level was calculated using the 2^−ΔΔCT^ algorithm, in which ΔCT = the CT of the target gene minus the CT of *EF-1*α (the internal control), and ΔΔCT = ΔCT of any sample minus calibrator, which was the measurement average in stage I (Livak and Schmittgen, 2001). The expression level among different developmental stages was compared using one-way ANOVA, in conjunction with Duncan's test for *post-hoc* multiple comparisons, in which a significant difference was identified at *P* < 0.05. Prior to parametric tests, the assumptions of data normality and homogeneity of variance were verified by Shapiro-Wilk's test and Levene's test. All primers used for qPCR are listed in Table 3.

**Table 3:** Primers used for qPCR in the present study.

**Gene**	**Forward primer (5'-3')**	**Reverse primer (5'-3')**
*dLp/HDL-BGBP*	AACTGACTTCCTGCACCTCG	AGCTGTTGGTGAGAGTGACG
*MTP-ABC*	AGGATGCCAAGTATGCCAACT	AAACACACTAAGCCCACCCG
*MTP-AB*	TGATCCGAGGAGGAAAGGAG	AAAAAGGTGAGGAAGACCAAGAG
*MTP-B*	TGCTCACCATCACTCTCTCGT	CCTGCTGACAAAGGGTTCCA
*MTP-C*	ACTGCTTTCCTCACCCTTGG	ATAGCGTCCCCACCTCAAAC
*EF-1α*	GGCCAGGTTCAACGAGATCA	TCTTCTGCTTGCTCCACCAG

## Results

The *dLp/HDL-BGBP* cDNA was 14,787 bp in length in *S. paramamosain*, sharing 99.94% identity with the Illumina sequencing data (MT776010.1), and contained an open reading frame (ORF) of 14,496 bp enclosed by a 5′ UTR of 42 bp and 3′ UTR of 249 bp (MZ198223). The ORF was predicted to encode a protein of 4,831 amino acids (aa), including a 16 aa signal peptide, and the deduced protein was calculated to be 534.85 kDa in molecular mass. Two furin cleavage sites were predicted to be located at amino acid residues 2,990–2,993 (RAKR) and 4,096–4,099 (RVRR), resulting in an N-terminal fragment of 2,993 aa, central fragment of 1106 aa, and C-terminal fragment of 739 aa, of which molecular masses were estimated as 328.21, 126.70, and 80 kDa, respectively. LPD_N and unknown function (DUF) 1943 domain architectures were predicted to be located at the N-terminal fragment. *S. paramamosain* dLp/HDL-BGBP shared 63.43% identity with other decapodan dLp/HDL-BGBPs and was conserved in the structure and cleavage pattern (Supplementary Figure 1).

In hemolymph, 14 peptide fragments matched to *Portunus trituberculatus* apoLp of 1,253 aa (A0A5B7CQW6), with sequence coverage of 12.7%, and shared 87% identity with the *S. paramamosain* dLp/HDL-BGBP sequence from amino acid residue 99 to 1,333. Further, 31 peptide fragments matched to *P. trituberculatus* BGBP of 3,444 aa (A0A5B7CMF4), with sequence coverage of 9.3%, and shared 89% identity with the *S. paramamosain* dLp/HDL-BGBP sequence from amino acid residue 1,387 to 4,831. Based on the furin cleavage sites (amino acid residues 1,603–1,607 and 2,708–2,712), 15, 12, and 4 fragments were specifically matched to N-terminal, central, and C-terminal regions of BGBP, respectively. The peptide fragments identified in *S. paramamosain* plasma are listed in the Supplementary Table.

Three *MTP* cDNA fragments were identified in *S. paramamosain* and were named *MTP-A, MTP*-*B*, and *MTP*-*C*. The lengths were 2,905, 2,897, and 3,088 bp, respectively, and *MTP-A, MTP-B*, and *MTP-C* varied in their 5′ end regions and shared a 2,711 bp identical sequence (MZ198220, MZ198221, and MZ198222). *MTP-A* consisted of a 5′ UTR of 148 bp, a 3′ UTR of 111 bp, and an ORF of 2,646 bp, which was predicted to encode an 881 aa protein, which included a 20 aa signal peptide followed by a LPD_N domain architecture. Compared with *MTP-A*, there were additional 24 nucleotides at the 5′ end of the *MTP*-*B* ORF, and this transcript was translated into an 889 aa protein, including a 19 aa signal peptide. *MTP*-*C*, sharing 99.96% identity with the Illumina sequencing data (MT776011.1), contained a 5′ UTR of 217 bp and an ORF of 2,760 bp, which was predicted to encode a 919 aa protein without a signal peptide. The *S. paramamosain* MTPs shared 79.49% identity with other decapodan MTPs, which supported the presence of MTP isoforms (Supplementary Figure 2).

**Figure 1:**
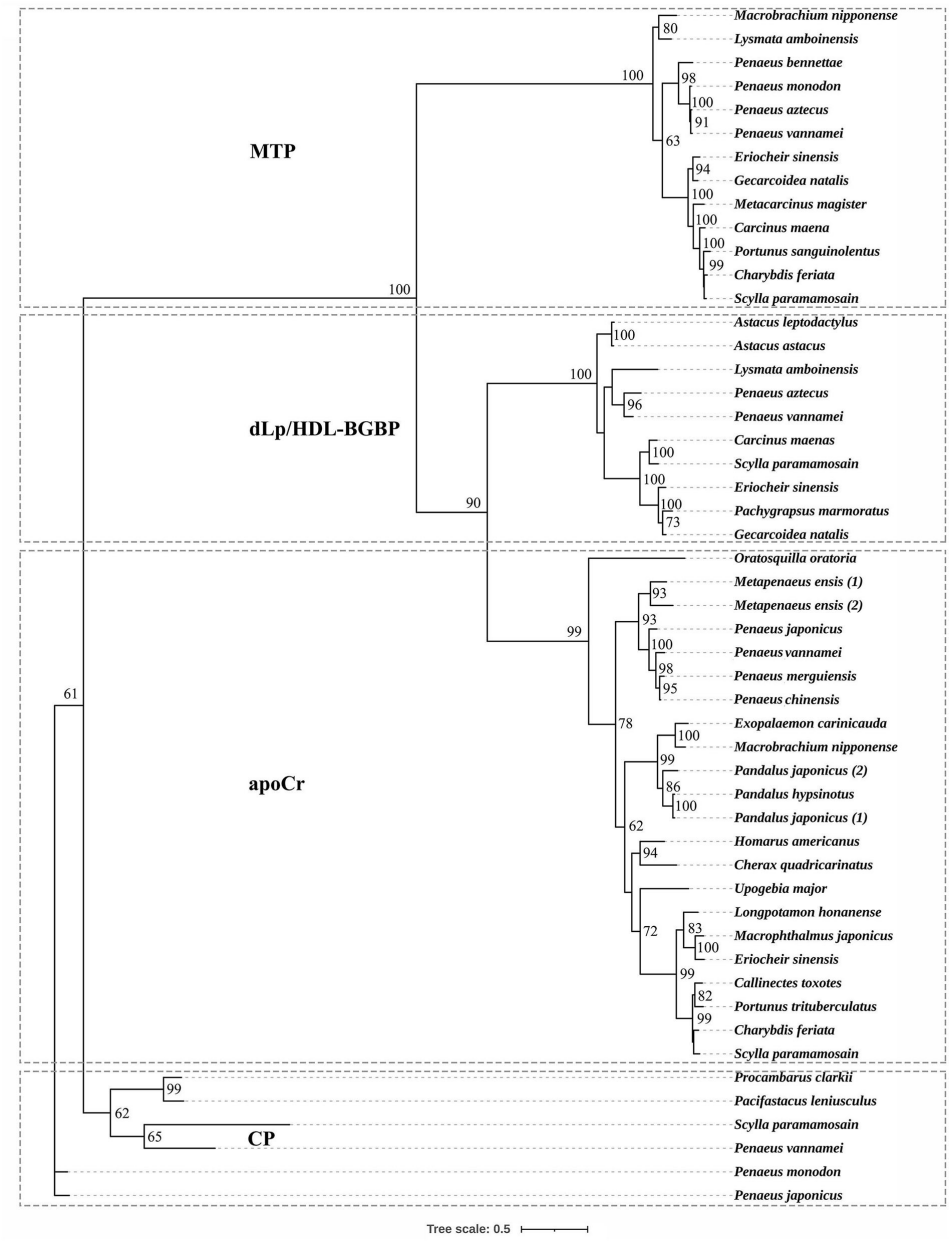
Phylogenetic tree of large lipid transfer proteins in decapodan species, using the maximum likelihood method. The tree topology was based on the alignment of the conserved amino acid sequence of the LPD_N domain. The numbers at each node indicate bootstrap values and bootstrap values < 50 are not provided. apoCr, apolipocrustacein; CP, clotting protein; dLp/HDL-BGBP, precursor of discoidal lipoprotein and beta-1,3-glucan binding protein; MTP, microsomal triglyceride transfer protein. Detailed information of LLTPs is listed in the Supplementary Table.

Based on the phylogenetic tree, decapodan LLTPs were divided into four groups, which consisted of apoCrs, dLp/HDL-BGBPs, MTPs, and CPs (Figure 1). The apoCr group was closely related to the dLp/HDL-BGBP group, subsequently to the MTP group, and distally to the CP group. In the dLp/HDL-BGBP group, *S. paramamosain* dLp/HDL-BGBP was closely clustered with other crab species, followed by shrimp/prawn and crayfish species. A similar case was observed in the MTP group in which *S. paramamosain* MTP was clustered closely with other crab species, followed by shrimp/prawn species.

**Figure 2:**
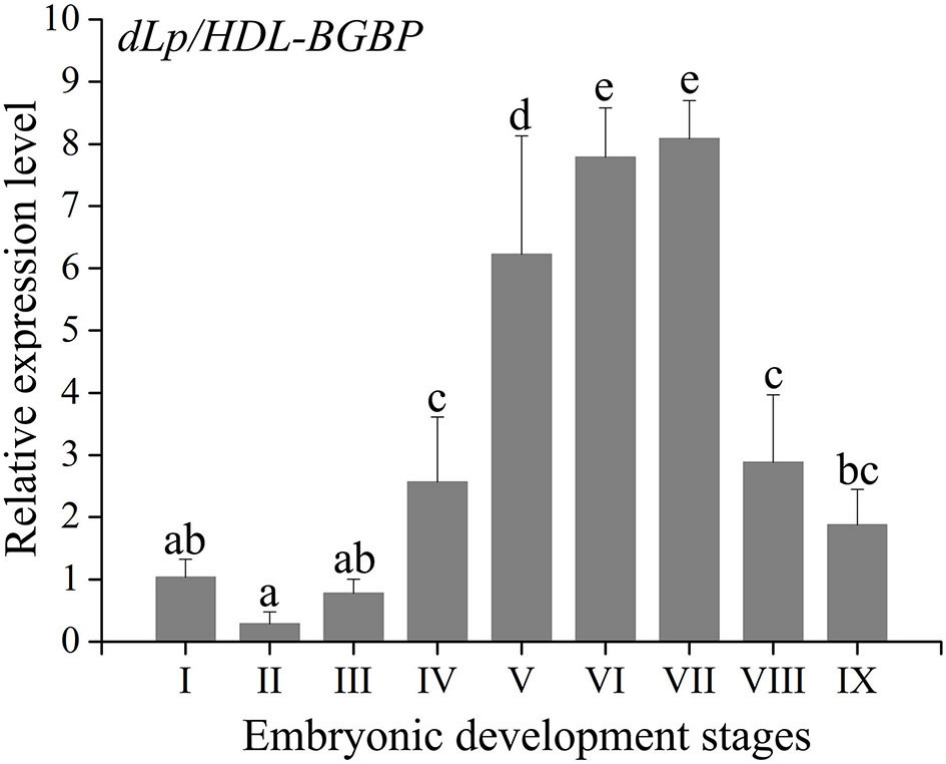
Expression profile of *dLp/HDL-BGBP* during embryonic development in *Scylla paramamosain*. dLp/HDL-BGBP, precursor of discoidal lipoprotein and beta-1,3-glucan binding protein. The relative expression level of *dLp/HDL-BGBP* was determined in blastocyst (I), gastrula (II), nauplius (III), early and late appendage formation (IV–V), early, middle, and late eye pigment formation (VI–VIII), and prehatching (IX) stages of embryos by qPCR, and *EF-1*α was the endogenous control. The same letters indicate no significant difference in expression level among embryonic development stages, according to one-way ANOVA in combination with Duncan's test for *post-hoc* multiple comparisons. A significant difference was identified at *P* < 0.05.

**Figure 3:**
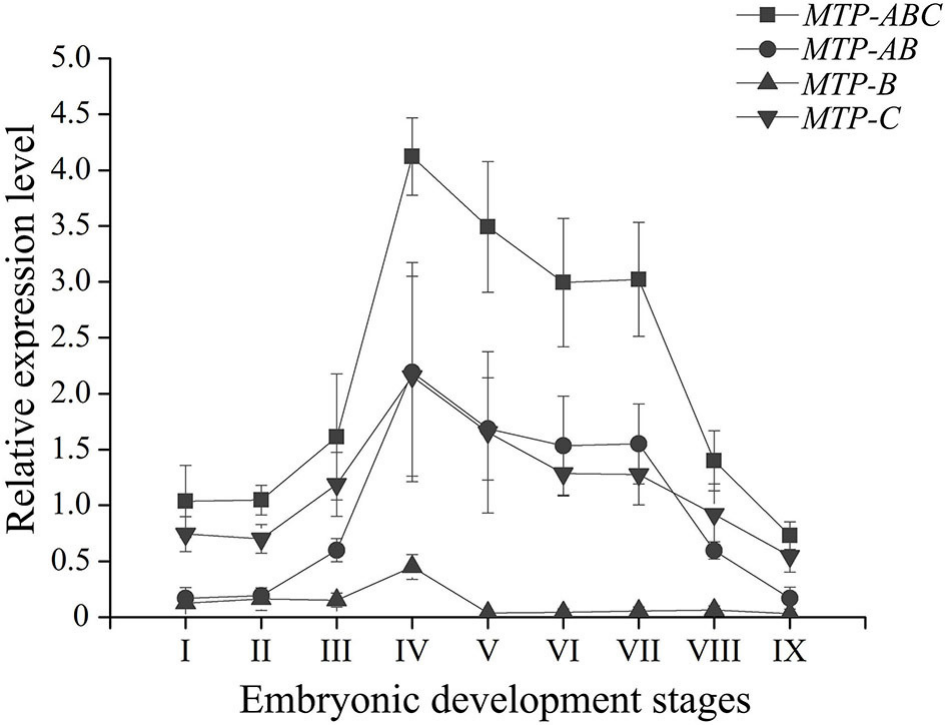
Expression profile of *MTP* transcript variants during embryonic development in *Scylla paramamosain*. MTP, microsomal triglyceride transfer protein. The relative expression level of *MTP* transcript variants was determined in blastocyst (I), gastrula (II), nauplius (III), early and late appendage formation (IV–V), early, middle, and late eye pigment formation (VI–VIII), and prehatching (IX) stages of embryos by qPCR, and *EF-1*α was the endogenous control.

During embryo development, the expression level of *dLp/HDL-BGBP* significantly increased from stage IV (mean ± SD = 0.29 ± 0.19), peaked in stage VI (mean ± SD = 7.79 ± 0.79), and VII (mean ± SD = 8.09 ± 0.61), and decreased in stage VIII (mean ± SD = 2.88 ± 1.09) and IX (mean ± SD = 1.87 ± 0.58) (*F* = 45.31, *P* < 0.01) (Figure 2). The *MTP* expression level was significantly higher in stages IV–VII (mean ± SD = 4.12 ± 0.35, 3.50 ± 0.58, 3.00 ± 0.57, 3.01 ± 0.52) than that in stages I–III (mean ± SD = 1.04 ± 0.32, 1.05 ± 0.13, 1.61 ± 0.57), and VIII–IX (mean ± SD = 1.40 ± 0.27, 0.73 ± 0.12) (*F* = 35.55, *P* < 0.01), using primer *MTP-ABC*, which amplified an identical sequence of *MTP-A, MTP-B*, and *MTP-C* (Figure 3, Table 4). The *MTP-C* mainly accounted for the *MTP* gene expression in stages I–III (mean ± SD = 0.74 ± 0.16, 0.70 ± 0.13, 1.19 ± 0.29) and VIII–IX (mean ± SD = 0.92 ± 0.27, 0.55 ± 0.15). *MTP-A* and *MTP-B* were mainly expressed in stages IV–VII (mean ± SD = 2.19 ± 0.98, 1.69 ± 0.46, 1.54 ± 0.44, 1.55 ± 0.36), using primer *MTP-AB*, which amplified an identical sequence for *MTP-A* and *MTP-B*, and the expression level was comparable to that of *MTP-C* (mean ± SD = 2.16 ± 0.89, 1.66 ± 0.72, 1.29 ± 0.20, 1.28 ± 0.27). The *MTP-B* expression level was maintained at a low-level throughout embryonic development, especially in stages VI–IX. The detailed information of *MTP* expression data is provided in Table 4.

**Table 4:** Expression levels of MTP transcripts during embryo development.

**Stage**	***MTP-ABC***	***MTP-AB***	***MTP-B***	***MTP-C***
I	1.04 ± 0.32 ab C	0.17 ± 0.09 a A	0.13 ± 0.02 abc A	0.74 ± 0.16 ab B
II	1.05 ± 0.13 ab C	0.19 ± 0.02 a A	0.16 ± 0.10 c A	0.70 ± 0.13 ab B
III	1.61 ± 0.57 b B	0.60 ± 0.10 a A	0.15 ± 0.06 bc A	1.19 ± 0.29 abc B
IV	4.12 ± 0.35 d C	2.19 ± 0.98 c B	0.45 ± 0.11 d A	2.16 ± 0.89 d B
V	3.50 ± 0.58 c C	1.69 ± 0.46 bc B	0.04 ± 0.01 a A	1.66 ± 0.72 cd B
VI	3.00 ± 0.57 c C	1.54 ± 0.44 b B	0.04 ± 0.03 a A	1.29 ± 0.20 bc B
VII	3.01 ± 0.52 c C	1.55 ± 0.36 b B	0.06 ± 0.04 a A	1.28 ± 0.27 bc B
VIII	1.40 ± 0.27 b D	0.60 ± 0.08 a B	0.07 ± 0.04 ab A	0.92 ± 0.27 ab C
IX	0.73 ± 0.12 a C	0.17 ± 0.10 a A	0.03 ± 0.01 a A	0.55 ± 0.15 a B

*MTP, microsomal triglyceride transfer protein. The relative expression level of MTP transcript variants was determined in blastocyst (I), gastrula (II), nauplius (III), early and late appendage formation (IV–V), early, middle, and late eye pigment formation (VI–VIII), and prehatching (IX) stages of embryos by qPCR, and EF-1α was the endogenous control. The same lower-case letters indicate no significant difference between embryonic development stages and same capital letters indicate no significant difference between MTP transcript variants, according to one-way ANOVA in combination with Duncan's test for post-hoc multiple comparisons. A significant difference was identified at P < 0.05*.

## Discussion

*Scylla paramamosain* dLp/HDL-BGBP shared a high homology, had two conservative domain architectures, and displayed a similar cleavage pattern with that of other decapodan species (Supplementary Figure 1). In *A. leptodactylus*, dLp/HDL-BGBP has been first proven as the common precursor of HDL-BGBP and dLp, following the isolation of dLp in hemolymph (Stieb et al., 2008, 2014). As a result of two cleavages at amino acid residue 3,047–3,050 and 4,155–4,158, the central fragment forms HDL-BGBP and N- and C-terminal fragments compose dLp, which are the large and small subunits of dLp, respectively (Stieb et al., 2008, 2014). Although transcriptome data do indicate dLp/HDL-BGBP synthesis in the hepatopancreas, implausibly, the same effort to isolate dLp in hemolymph failed for *Astacus astacus, Macrobrachium rosenbergii*, and several other species (Stieb et al., 2008, 2014). In the present study, peptide fragments of HDL-BGBP and dLp large and small subunits were all identified in hemolymph, which confirmed that both were secreted into hemolymph in *S. paramamosain*. The failed dLp isolation in previous studies using KBr density gradient ultra-centrifugation was probably due to a low-level or lack of lipidation of dLp. A possible explanation for the highly lipidated dLp exclusively observed in *A. leptodactylus* is that the sampled animals might be in a different physiological state.

Lipoproteins in discoidal shape are also observed in other animal phyla. In the polychaete *Nereis virens*, a discoidal lipoprotein was isolated from coelomic fluid and found to have a critical role in lipid transfer to spermatozoa (Schenk et al., 2006; Schenk and Hoeger, 2010). The *N. virens* dLp comprises two subunits with molecular masses of 247 and 85 kDa, which are similar to the *A. leptodactylus* dLp large subunit of 240 kDa and dLp small subunit of 80 kDa, and the lipid composition of *N. virens* dLp is also similar to that of decapodan lipoproteins (Schenk et al., 2006; Stieb et al., 2008). Nonetheless, the scaffolding protein of *N. virens*, dLp, is still unknown. In insects and vertebrates, non-LLTP superfamily members are involved in discoidal shape lipoprotein formation, namely apoLp III and apoA-I, which are both exchangeable components of lipoproteins (Koppaka, 2001; Weers and Ryan, 2003). These findings indicate that the origins of scaffolding proteins in discoidal shape are likely to vary among animals.

The *dLp/HDL-BGBP* expression level was consecutively increased from the early appendage formation stage to the eye pigment-formation stage in *S. paramamosain* embryos. The increase in *dLp/HDL-BGBP* expression level indicated that HDL-BGBP, known as the major circulatory lipid carrier, probably also functioned as a scaffolding protein for lipid transfer during embryonic development, which was robustly supported by its negative relationships with lipid contents of yolk sacs in this species. According to anatomic and histologic observations, Chen (2005) demonstrates that lipid droplets in the yolk sac are initially degraded in the gastrula stage, which is dramatically increased from the early appendage-formation stage, and that lipid droplets are rarely observed up to the late eye pigment-formation stage. Consistent findings have been demonstrated in *Callinectes sapidus* (Walker et al., 2003). Specifically, anti-HDL/BGBP immunoreactivity can be detected in lateral basophilic cuboidal cells of embryos that are adjacent to the yolk mass, and the HDL/BGBP expression level continuously increases from the early appendage-formation stage to the prehatching stage based on ELISA assays, which suggests that HDL/BGBPs transport lipids from the yolk sac to embryonic tissues (Walker et al., 2003). Strikingly, the same has been proven in other oviparous animals, for example, apoLp-II/I in insects and apoBs in reptiles, birds, and fish being the major lipid carriers in the circulatory system and transporting lipid from the yolk sac to embryonic tissues (Thompson and Speake, 2002; Tsuchida et al., 2010; Schneider, 2016). These findings suggest that the major lipid carrier during embryonic development is identical to that in the post-embryonic circulatory system in most oviparous animals.

Three *MTP* transcript variants were identified in *S. paramamosain*. Among the variants, *MTP-C* has been previously assembled from hepatopancreas transcriptome data (Zeng et al., 2021), and *MTP-A* and *MTP-B* were newly identified in this species. They were predicted to encode three MTP isoforms which were different merely in their N-terminal region. According to transcriptome data, the three types of *MTP* transcript variants and/or MTP protein isoforms appear to be widely distributed in decapodan species (Supplementary Figure 2). In fact, multiple *MTP* transcript variants and/or MTP protein isoforms are extensively observed in invertebrates and vertebrates (Mohler et al., 2007; Suzuki and Swift, 2016; Suzuki et al., 2016; Khan et al., 2017). For example, three *MTP* transcript variants were identified in the salmon louse *Lepeophtheirus salmonis*, and these were putatively translated into two MTP isoforms (Khan et al., 2017). In vertebrates, there are three *MTP* transcript variants that have been identified both in humans and mice, which turned out to encode two MTP isoforms in mice but only a single translation product in humans (Mohler et al., 2007; Suzuki and Swift, 2016; Suzuki et al., 2016). In agreement with our findings, sequence differences between transcript variants of *MTP* genes are also exclusively present in the 5′ end region in these species (Mohler et al., 2007; Suzuki and Swift, 2016; Suzuki et al., 2016; Khan et al., 2017). These findings indicated that transcription of the *MTP* gene might be conserved across animal phyla.

One remaining question is the importance of multiple transcripts of the *MTP* gene in animals. As mentioned, three *MTP* transcript variants (*MTP-A, MTP-B*, and *MTP-C*) are present in mice and humans (Suzuki and Swift, 2016; Suzuki et al., 2016). In mice, *MTP-A* and *MTP-C* are both translated into MTP-A, which is dedicated to lipoprotein assembly, and its expression level is regulated by distinct promoters. Further, the translation efficiencies of these genes are differentiated by their 5′ UTRs; *MTP-B* shares an identical mRNA precursor with *MTP-C* and is translated into MTP-B, which is involved in lipid droplet maturation (Mohler et al., 2007; Suzuki et al., 2016). Except for the fact that *MTP-B* does not encode a protein, findings in humans are consistent with those of mice (Suzuki and Swift, 2016). These findings in vertebrates indicate that multiple transcripts could be involved in expression regulation and/or functional diversity, which is species-specific. In addition, more than one APO family member has been identified in the circulatory systems of many invertebrate species, and for all, lipidation is mediated by MTP, similar to that for VTGs (Sellers et al., 2005; Palm et al., 2012; Wu et al., 2013; Zeng et al., 2021). Consequently, multiple transcripts of the *MTP* gene might be partially related to distinct scaffolding protein lipidation. Nonetheless, studies regarding MTP expression and function are still in their infancy, especially for invertebrates.

The *MTP* gene expression varies highly throughout embryonic development in *S. paramamosain*. *MTP-C* accounts for ~70% of *MTP* mRNA in the early development stage of embryos (blastocyst to nauplius), as well as in the final stage (prehatching), and its expression level was found to be comparable to that of *MTP-A* from the early appendage-formation stage to the late eye pigment-formation stage, of which the expression level was extremely low in blastocyst and gastrula stages. *MTP-B* was determined to be expressed at a low level from the blastocyst stage to the nauplius stages, and the expression level was nearly negligible after the late appendage-formation stage. These distinct expression profiles might be related to different biological processes. As mentioned, besides lipoprotein assembly, MTP is also dedicated to lipid droplet maturation (Mohler et al., 2007, Love et al., 2015). Lipid droplets are involved in many physiologic processes in the embryo, such as energy storage, histone sequestration, and antibacterial responses (Anand et al., 2012; Li et al., 2012; Welte, 2015b; Aizawa et al., 2019). The lipid droplets in embryonic tissues are constructed with lipids that are transported from the yolk sac in oviparous animals (Heras et al., 2000; Speake and Thompson, 2000; Yamahama et al., 2008; Miyares et al., 2014; Schneider, 2016). Undoubtedly, lipoprotein assembly in the yolk sac occurs almost simultaneously with lipid droplet maturation in embryonic tissues during embryonic development. The role in lipoprotein assembly might be played by MTP-A, of which the expression level was extremely low in the early stage and the ending stage of embryonic development, during which the lipid transfer activity was low that was evidenced by the expression profile of *dLp/HDL-BGBP* and previous histologic findings (Chen, 2005). Meanwhile, the role in lipid droplet maturation alternatively appeared to be played by MTP-C. In addition, an explanation of expression regulation could not be delineated, in terms of multi-transcript expression in *S. paramamosain*. Nonetheless, the specific roles of *MTP* transcripts or their translation during embryo development require further verification in *S. paramamosain*.

## Data Availability Statement

The datasets presented in this study can be found in online repositories. The names of the repository/repositories and accession number(s) can be found in the article/Supplementary Material.

## Author Contributions

XZ, ZZ, and YW conceived the study and wrote and edited the manuscript. XZ and LL collected samples. XZ, LL, DZ, JZ, YF, HW, and CL participated in acquisition, analysis, and interpretation of data. All authors reviewed, contributed, and approved the manuscript.

## Conflict of Interest

The authors declare that the research was conducted in the absence of any commercial or financial relationships that could be construed as a potential conflict of interest.

## Publisher's Note

All claims expressed in this article are solely those of the authors and do not necessarily represent those of their affiliated organizations, or those of the publisher, the editors and the reviewers. Any product that may be evaluated in this article, or claim that may be made by its manufacturer, is not guaranteed or endorsed by the publisher.
