# Supplementary material for: dLp/HDL-BGBP and MTP Cloning and Expression Profiles During Embryonic Development in the Mud Crab Scylla paramamosain
Source: Front Physiol. 2021 Aug 19;12:717751. doi: 10.3389/fphys.2021.717751 (PMC8416765; doi:10.3389/fphys.2021.717751)
Supplement: Supplementary file 1 [file Data_Sheet_1.doc]

| **Table 1** Decapodan LLTPs with cDNA sequence support used in present study. | | |
| --- | --- | --- |
| Protein name | Species | Accession no. |
| dLp/HDL-BGBP | *Pontastacus leptodactylus* | AHJ78589.1 |
| dLp/HDL-BGBP | *Astacus astacus* | AHK23026.1 |
| apoCr | *Scylla paramamosain* | FJ812090.1 |
| apoCr | *Charybdis feriata* | AAU93694.1 |
| apoCr | *Eriocheir sinensis* | AGM75775.1 |
| apoCr | *Macrophthalmus japonicus* | QEJ88142.1 |
| apoCr | *Portunus trituberculatus* | AAX94762.1 |
| apoCr | *Callinectes toxotes* | QGA73526.1 |
| apoCr | *Longpotamon honanense* | AKI23633.1 |
| apoCr | *Upogebia major* | BAF91417.1 |
| apoCr | *Penaeus merguiensis* | Q6RG02.2 |
| apoCr | *Penaeus vannamei* | AAP76571.2 |
| apoCr | *Penaeus japonicus* | BAB01568.1 |
| apoCr 1 | *Metapenaeus ensis* | AAM48287.1 |
| apoCr 2 | *Metapenaeus ensis* | AAT01139.1 |
| apoCr | *Penaeus chinensis* | ABC86571.1 |
| apoCr | *Pandalus hypsinotus* | BAD11098.1 |
| apoCr 1 | *Pandalus japonicus* | ACU51164.1 |
| apoCr 2 | *Pandalus japonicus* | AHD26978.1 |
| apoCr | *Oratosquilla oratoria* | ALI16501.1 |
| apoCr | *Macrobrachium nipponense* | AJP60219.1 |
| apoCr | *Exopalaemon carinicauda* | AFM82474.1 |
| apoCr | *Homarus americanus* | ABO09863.1 |
| apoCr | *Cherax quadricarinatus* | AAG17936.1 |
| CP | *Penaeus monodon* | Q9U572.1 |
| CP | *Pacifastacus leniusculus* | AAD16454.1 |
| CP | *Penaeus vannamei* | ROT65756.1 |
| CP | *Procambarus clarkii* | AYD41596.1 |
| CP | *Penaeus japonicus* | ABK59925.1 |
| CP | *Scylla paramamosain* | MG668645.1 |

| **Table 2** *dLp/HDL-BGBP* and *MTP* retrieved from transcriptome data of decapodan species in present study. | | | |
| --- | --- | --- | --- |
| **Species** | **Project no.** | ***dLp/HDL-BGBP*** | ***MTP*** |
| *Carcinus maenas* | PRJNA255867 | GBXE01107331.1 | GBXE01069344.1 |
| *Charybdis feriata* | PRJNA415670 |  | GGFD01039627.1 |
| *Eriocheir sinensis* | PRJNA350735 | GFBK01029246.1 | GFBK01009864.1, GFBK01009863.1 |
|  | PRJNA386404 |  | GGQO01003334.1 |
| *Gecarcoidea natalis* | PRJNA412130 | GFXJ01000003 | GFXJ01001542.1 |
| *Lysmata amboinensis* | PRJNA548450 | GHOJ01034342.1 | GHOJ01037648.1, GHOJ01037649.1 |
| *Macrobrachium nipponense* | PRJNA278700 |  | GCVG01016373.1 |
| *Metacarcinus magister* | PRJNA550297 |  | GHOI01003966.1 |
| *Pachygrapsus marmoratus* | PRJNA605968 | GIJU01049032.1 |  |
| *Penaeus aztecus* | PRJNA326516 | GEUA01065821.1 | GEUA01062825.1 |
| *Penaeus vannamei* | PRJNA326516 | GETZ01046384.1 |  |
|  | PRJNA323362 |  | GETD01040558.1, GETD01040556.1 |
|  | PRJNA411958 |  | GGUK01025132.1 |
| *Penaeus bennettae* | PRJNA509986 |  | GHDJ01007130.1 |
| *Penaeus monodon* | PRJNA421400 |  | GGLH01009918.1, GGLH01007262.1 |
| *Portunus sanguinolentus* | PRJNA415705 |  | GFZC01051025.1 |

| **Table 3** dLp/HDL-BGBP peptide fragments identified in *S. Paramamosain* hemolymph. | | | | |
| --- | --- | --- | --- | --- |
| Sequence | Length | Mass | Proteins | position |
| dLp large subunit |  |  |  |  |
| DGPVPTSTEQQFTR | 14 | 1561.7372 | A0A5B7CQW6 | 167-180 |
| GLEYPVLSK | 9 | 1004.5542 | A0A5B7CQW6 | 232-240 |
| VYADTTDTHSR | 11 | 1264.5684 | A0A5B7CQW6 | 241-251 |
| KFLVDAMPLVGTAAAAAVVR | 20 | 1999.1289 | A0A5B7CQW6 | 252-271 |
| FLVDAMPLVGTAAAAAVVR | 19 | 1871.0339 | A0A5B7CQW6 | 253-271 |
| CGEDSGVQQVMR | 12 | 1364.5813 | A0A5B7CQW6 | 335-246 |
| RVEAQLGSGCR | 11 | 1231.6092 | A0A5B7CQW6 | 347-357 |
| VEAQLGSGCR | 10 | 1075.508 | A0A5B7CQW6 | 348-357 |
| CYTENNDMEVR | 11 | 1429.5602 | A0A5B7CQW6 | 390-400 |
| LTSEGVNQVGSFVWTHLTNLQESAAPGK | 28 | 2969.4883 | A0A5B7CQW6 | 456-483 |
| VLLGEELLANK | 11 | 1197.6969 | A0A5B7CQW6 | 488-498 |
| LFGNDVHYR | 9 | 1119.5461 | A0A5B7CQW6 | 623-631 |
| VSSTMHTSTALEGK | 14 | 1447.6977 | A0A5B7CQW6 | 739-752 |
| TEFIDMETK | 9 | 1112.506 | A0A5B7CQW6 | 772-780 |
| NSQQATLSVK | 10 | 1074.5669 | A0A5B7CMF4 | 68-77 |
| LSLAPGQHDVDLALK | 15 | 1575.8621 | A0A5B7CMF4 | 190-204 |
| TQLEVVPDMEATVK | 14 | 1558.7913 | A0A5B7CMF4 | 207-220 |
| FTSQFEVTR | 9 | 1113.5455 | A0A5B7CMF4 | 285-293 |
| IWSTYSFGSLK | 11 | 1287.6499 | A0A5B7CMF4 | 294-304 |
| GSFSFTHK | 8 | 909.43447 | A0A5B7CMF4 | 330-337 |
| IENNNLTTSAK | 11 | 1203.6095 | A0A5B7CMF4 | 338-348 |
| ISAEVEYKPGSK | 12 | 1306.6769 | A0A5B7CMF4 | 511-522 |
| YGSQEVDLR | 9 | 1065.5091 | A0A5B7CMF4 | 760-768 |
| KIEVTGTQHLR | 11 | 1280.7201 | A0A5B7CMF4 | 811-821 |
| VTLNITTPFAELR | 13 | 1473.8191 | A0A5B7CMF4 | 883-895 |
| EFAFSAYGR | 9 | 1046.4821 | A0A5B7CMF4 | 1356-1364 |
| YTQESITLK | 9 | 1081.5655 | A0A5B7CMF4 | 1365-1373 |
| LIGSDLSFDLR | 11 | 1234.6558 | A0A5B7CMF4 | 1577-1587 |
| LNMSGSVNR | 9 | 976.47601 | A0A5B7CMF4 | 1596-1604 |
| HDL-BGBP |  |  |  |  |
| MEEVSFEIK | 9 | 1110.5267 | A0A5B7CMF4 | 1640-1648 |
| MEEVSFEIKR | 10 | 1266.6278 | A0A5B7CMF4 | 1640-1649 |
| SELQGWEFLALAGR | 14 | 1575.8045 | A0A5B7CMF4 | 1684-1697 |
| IESNSSNFK | 9 | 1024.4825 | A0A5B7CMF4 | 1760-1768 |
| VGPESSYR | 8 | 893.4243 | A0A5B7CMF4 | 2060-2067 |
| INIDISDLNPR | 11 | 1268.6725 | A0A5B7CMF4 | 2184-2194 |
| RQATVDYVR | 9 | 1106.5833 | A0A5B7CMF4 | 2446-2454 |
| SQFNLSFTAPSGK | 13 | 1382.683 | A0A5B7CMF4 | 2472-2484 |
| LDSELNTQQR | 10 | 1202.5891 | A0A5B7CMF4 | 2553-2562 |
| LNDFVFEVHNEFVR | 14 | 1763.8631 | A0A5B7CMF4 | 2570-2583 |
| YDDGYYIK | 8 | 1035.4549 | A0A5B7CMF4 | 2584-2591 |
| IFTLGYGEEK | 10 | 1155.5812 | A0A5B7CMF4 | 2616-2625 |
| dLp small subunit |  |  |  |  |
| LMSPFTGQHSAR | 12 | 1330.6452 | A0A5B7CMF4 | 2698-2709 |
| ANATILPGHLIGNK | 14 | 1417.8041 | A0A5B7CMF4 | 2915-2928 |
| ATVTTPLESFK | 11 | 1192.634 | A0A5B7CMF4 | 3289-3299 |
| APYELAYK | 8 | 953.48583 | A0A5B7CMF4 | 3414-3421 |
