# Supplementary figures and images for: dLp/HDL-BGBP and MTP Cloning and Expression Profiles During Embryonic Development in the Mud Crab Scylla paramamosain
Source: Front Physiol. 2021 Aug 19;12:717751. doi: 10.3389/fphys.2021.717751 (PMC8416765; doi:10.3389/fphys.2021.717751)

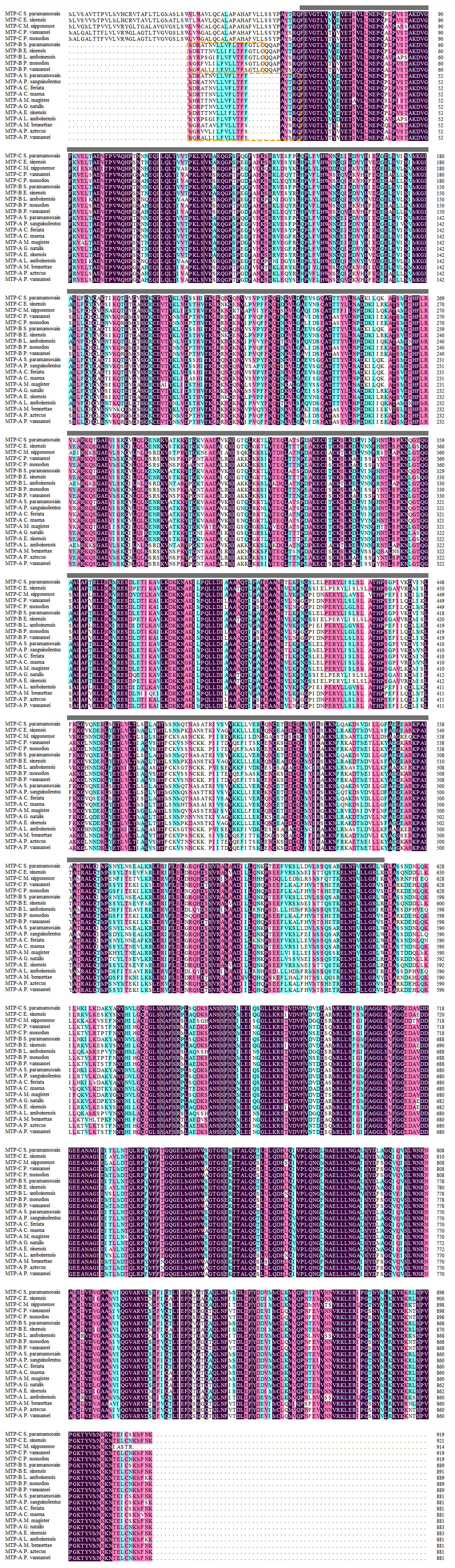

Supplement: Supplementary file 3 [file Image_2.JPEG]
